# Shining light inside the tunnel: using photovoice as a strategy to define the needs for health promotion among families of low socioeconomic status

**DOI:** 10.1080/17482631.2018.1542909

**Published:** 2018-11-19

**Authors:** Lotte Prevo, Kelly Stessen, Stef Kremers, Marjolein Wassenberg, Maria Jansen

**Affiliations:** a NUTRIM, Department of Health Promotion, Maastricht University, Maastricht, The Netherlands; b Department of Society, Municipal Government of Vaals, Vaals, The Netherlands; c Department of Knowledge and Innovation, Public Health Service Southern Limburg, Heerlen, The Netherlands; d CAPHRI, Department of Health Services Research, Maastricht University, Maastricht, The Netherlands; e Academic Collaborative Center for Public Health, Public Health Service Southern Limburg, Heerlen, The Netherlands

**Keywords:** Low socioeconomic status families, needs, opportunities, photovoice, community approach, health promotion

## Abstract

Purpose: This study aimed to identify opportunities to improve the current health and social situation of low socioeconomic status (SES) families and to gain a better understanding of the main needs regarding health promotion. Low-SES families were approached to participate in a photovoice study. Method: The study took place in the municipality of Vaals, which is located in the southernmost part of the Netherlands. A diverse group of ten people from eight different families took about 150 photographs within their community on topics they considered important for their health and quality of life. This was followed by individual interviews and a focus group interview. Results: Four main needs were identified: meeting each other, helping each other, feeling safe and being mobile. The photographs showed that health-related themes had low priority for these families. Conclusion: The low-SES families focused on upstream factors relating to independence, self-resilience and a sense of belonging, to help them cope with their current situation. This study represents a first step towards the development of a community approach to health promotion in low-SES families.

## Background

For many years, intervention programmes were in place trying to reduce the health gap between low socioeconomic status (SES) populations and middle-to-high SES populations (Marmot, Allen, Bell, Bloomer, & Goldblatt, 2012; Mullins, Blat, Gbarayor, Yang, & Baquet, 2012). However, intervention programmes that prove to be effective in a research setting but remain on the developers’ shelf, projects that stop after funding runs out, and interventions that do not appeal to the group for whom they were actually intended are frequently heard horror stories in health sciences. These issues tend to arise especially in programmes targeting these low-SES community groups, who seem to experience barriers to participation (Bonevski et al., 2014). According to Ball (2015) and Mullainathan and Shafir (2013), the living conditions of low-SES families may lead to tunnel vision, as they focus on their income difficulties (Ball, 2015; Mullainathan & Shafir, 2013). In view of the mental energy that needs to be devoted to issues that relate to being poor, low-SES individuals cannot afford to give their cognitive attention to any other goals they could act upon. They experience the effort to adopt a healthy lifestyle as a cognitive burden (Ball, 2015).

In the past, health promotion professionals have developed well-intentioned health programmes for low-SES groups on the basis of what they thought these groups needed. In fact, they often completely misunderstood the fundamental issues in a low-SES community (Community Tool Box, 2016; Mitrofanova, 2005; Movisie, 2012a). This made it difficult to actually nudge low-SES families into active participation in programmes aimed at improving their health status (Van den Berg-Slagter, 2006). The developers’ lack of penetration into these communities has been a major barrier to the development and implementation of successful community-based health promotion programmes (Carlson, Engebretson, & Chamberlain, 2006).

Currently, greater emphasis is being placed on possibilities to include the target group in the process of developing and implementing health promotion programmes, based on what the community itself regards as important, in order to prevent mismatch failures (Movisie, 2012b; National Institute for Public Health and the Environment, 2017). The involvement of low-SES families should be supported from the very beginning of the whole programme planning process, to create a better understanding of the issues they perceive (Carlson et al., 2006; Kahraman, 2010; Scheerder, Van der Broucke, & Saan, 2003). Achieving this requires striking a balance between top-down processes initiated by the interventionists and bottom-up processes initiated by the practitioners who work with low-SES families, so as to ensure a tailored programme development and implementation process (Van Kann, Jansen, De Vries, De Vries, & Kremers, 2015). Working *with* instead of *for* low-SES families can create a greater ability to ensure the adoption, implementation and continuation of the programme (National Institute for Public Health and the Environment, 2017).

Generally speaking, low-SES people often have lower than average educational levels and illiteracy is more prevalent among them. For example, 19% of the Dutch low-SES population are low-literate, compared with an average of 11.9% of the total population in the Netherlands (Christoffels, Baay, Bijlsma, & Levels, 2016). This means it is a challenge for these people to verbally express themselves in research settings and to fill out questionnaires. Researchers find it hard to understand the needs of low-SES families and to give them a voice. One effective approach to gain more insights, and to get the low-SES families involved, may be the photovoice technique developed by Wang and Burris (1997). In a photovoice project, participants are instructed to make photographs of things that influence their lives, their situation or their health, which assists them to speak out through photography (Wang & Burris, 1997). In this study, photovoice enables low SES-families to express the strengths and weaknesses in their community, making it easier for professionals such as health promoters or policymakers to get an in-depth idea of the views of the families themselves (Merzel & D’Afflitti, 2003; Strack, Magill, & McDonagh, 2004; Wang & Burris, 1997). In the end, participating in a photovoice study can be an excellent instrument to build capacity for joining in community-based programmes that support low-SES populations in overcoming their socially disadvantaged situation (Carlson et al., 2006; Wang & Burris, 1997).

The present study acknowledges the importance of understanding low-SES families as a first step towards a community-based health promotion programme. These families are viewed as experts, showing the opportunities they see to enhance their situation, and explaining the real difficulties they experience in daily life. The photovoice technique elicits their views (Wang & Burris, 1997). It enables the low-SES families to sensitize professionals to their real experiences, and offers gateways to the most important aims for future change (Jones, Ingham, Cram, Dean, & Davies, 2013; Movisie, 2012a; Parker, 2003). Our study aimed to answer the following research questions: “What are the main difficulties that low-SES families experience in daily life?” and “Which opportunities do they mention themselves to improve their current health status?”

## Method

### Study design

This photovoice study used a narrative research design. The photovoice method uses photographs made by the participants themselves to enable them to tell their own stories (Wang & Burris, 1997). The personal-experience stories of the participants help to understand the opportunities they see to improve their position in daily life and to offer insight into the main difficulties that low-SES families experience (Creswell, Hanson, Plano Clark, & Morales, 2007). A constructivist paradigm was applied during the research activities of this study, focusing on the participants’ manifold experiences and truths (Bergman et al., 2012).

### Study setting

In the period from July 2015 to January 2016, a photovoice study was carried out in the municipality of Vaals, according to the Code of Conduct for Health Research of the Dutch Federation of Biomedical Scientific Societies and with the support of the Public Health Service Southern Limburg and Maastricht University. Vaals is a small town located in the southernmost part of the Netherlands, near the borders with Germany and Belgium. On January 2015, the municipality Vaals had 9694 residents, most of them people of Dutch origin (51.4%) or immigrants (44.4%) from Western countries, particularly Germany. The population of Vaals is ageing, as about 25% of the population are over 65 years old (Statistics Netherlands, 2015). The municipality of Vaals is regarded as a region whose residents have a poorer health status, a shorter life expectancy, more mental health issues, an unhealthy lifestyle and more chronic diseases, compared with the average Dutch population (Vermeer et al., 2014). The population can be characterized as moderate to low SES (Vermeer et al., 2014). Using data from Statistics Netherlands, we analyzed the characteristics of the low-SES families in Vaals. Having a low SES was found to be more common among the Western and non-Western immigrants, single-parent households, unemployed families and families living in rented housing and receiving housing and/or social benefits. Most low-SES families lived in the centre of Vaals, indicating a concentration of poverty in these neighbourhoods (Statistics Netherlands, 2015). The number of families living in poverty is relatively high. One in six families (N = 150) in Vaals lives below the poverty threshold, which is about twice as much as the average for the Netherlands, with one in eleven families living below the poverty threshold. The number of families with a permanently low income is also higher, at 5.2%, compared with the average of 3.3% for the Netherlands (Statistics Netherlands, 2017).

### Recruitment and study participants

Participants were included based on the following three inclusion criteria: (1) the family had to live around the poverty threshold, (2) at least one child had to be living at home and (3) they had to reside in the municipality of Vaals. The participants were recruited in the period from July 2015 till September 2015, using various channels: recruitment articles in local newspapers, an interview on local radio, organizations that have contacts with the target group, and a local intermediary in Vaals. The personal contacts of the intermediary turned out to be especially valuable in obtaining the participation of low-SES families. Ten participants could be recruited, aged between 18 and 65 years, from eight different families.

### Photovoice procedure and instruments

The data collection procedure of the study consisted of four phases. First, the ten participants were instructed, during individual meetings, about what they could photograph, how to make the pictures and that they could take an unlimited number of pictures. They were asked to take at least three pictures of things in their daily life that they perceived as positive, three of things that had a negative influence on their lives, three things they would like to change in the future and three that did not fit the other three theme’s categories. The underlying themes of these photos had to be health and quality of life. They were given two weeks to take the photographs. In the second phase, individual (recorded) semi-structured interviews were arranged to talk about and reflect the stories behind their photographs. The individual interviews were based on the PHOTO method developed by Larkin et al. (2007) to ensure a systematic interview procedure. For each picture that the participant had taken, five questions were asked. The participant was first asked to describe the picture, then to clarify what was happening in the picture, then to explain why they had taken the picture, to clarify what the picture told outsiders about their life, and finally to indicate the opportunities that the picture implied for the improvement of their lives. Third, a focus group interview with all participants was held to discuss the photographs and their stories. The main aim was to create space for a critical dialogue to be able to prioritize the themes portrayed in the photographs, to determine what themes should be most urgently addressed to improve their situation. Finally, an exhibition was organized in Vaals to present the most important findings, based on the prioritized themes, to citizens and network partners, especially the local policy makers. Visitors of the exhibition were asked to write down their thoughts about this study. This way, a first step in obtaining commitment from stakeholders to work together with the families on improving these themes, could be set.

### Data processing and analysis

The individual interviews and the focus group session were recorded and transcribed verbatim afterwards. These transcripts and the accompanying notes were analyzed with the Nvivo software to correctly organize the data (McDonnell, 2012). The analyses of the individual interviews resulted in four common themes that were discussed and confirmed by the participants during the focus group. For the exhibition, the participants selected the most important photographs relating to each theme, which helped with the integration of data and the prioritization of their messages (Wang, 1999). The transcripts of the individual interviews and the focus group session were used by the researcher to clearly describe the photographs and comments for each of the themes. By way of confirmation, the themes, photographs and comments for this exhibition were member-checked by the participants (Bugos et al., 2014).

## Results

### Background information participants

Ten participants from eight different families with at least one child to take care of, all living in Vaals, participated in the photovoice study. Participants were mostly female (80%). Half of the participants were married and half of them had two children at home to take care of. The majority of the participants had the Dutch nationality. The reasons that made them fall into poverty were reported to be: being a refugee, bankruptcy, divorce, being unfit for work, health issues of their children and being born into a poor family.

### Daily issues and opportunities

The photographs made by the participants (see Supplement 1 for an overview) and the stories they told reflected the variety of issues they experienced in daily life and the opportunities they saw for improving their situation. Almost 150 pictures were taken, reflecting things at home, in their community or across the border with Germany of Belgium, about their family and their wishes for the future. Four common themes could be defined during the individual and focus group interviews: meeting each other, helping each other, feeling safe and being mobile. The overall results are presented below.

#### Meeting each other

The most important theme mentioned by the participants was the opportunity for low-SES families in Vaals to meet each other. Participants reported being satisfied about the facilities in Vaals, the activities organized by the health and social organizations and the support funding they received from the municipal government.

*“Vaals does a lot to support people. For example, certain funds allow our children to do sports, they get support for their education and we can be members of the library.”*

On the other hand, participants said that it was often difficult for them to socialize. Families with children said they had contacts with other parents at school or at their children’s sports club, but that they felt isolated especially during holidays and at the weekend, when there were no organized activities. Families with older children in secondary school said that social contacts were mostly lacking and they felt they participated little in society.

*“For me it’s difficult to make new social contacts. I would really like to do so, but it might also be difficult because of my depression.”*

Another point raised by the participants was that they longed for a sense of belonging and trust. Participants mentioned that others made negative comments about low-SES families, which made them feel stigmatized.

*“For me it’s important to have a sense of belonging. That feels good and gives you a feeling of trust. It also feels good if my children feel involved in something. Because if my child is happy, then so am I.”*

Participants indicated that they did not have the opportunity to go to public places or events for example to drink a cup of coffee, as they did not have the money to do so. Most of the time they also needed to take their children with them, because childcare is too expensive.

*“I can’t go out to drink a cup of coffee. I don’t have the money to do so. How much does that cost? €2.50 or so? That’s a lot of money for me. Then I’d rather say to a friend of mine come to my place for coffee. But in the end you’re still at home and don’t meet others.”*

As a solution, participants would like to see a facility nearby, such as a community centre. This should be a place where people can meet each other, where all different ethnic and age groups in the community can come together, where activities can be organized and where people can help each other. This place should be facilitated by the municipality, but participants themselves were willing to take the initiative to make it a success.

*“Recently I was in Brunssum [a nearby municipality] for my therapy and there I saw a community centre. That’s ideal, Vaals also needs something like that. You could join different courses, social evenings and activities there.”*

#### Helping each other

The photographs and comments on the second theme, that of helping each other, indicated that the support from professionals employed by the local authorities of Vaals was appreciated, such as support from the housing corporation, the “Silent Poverty” foundation, and a local intermediary who supports people in becoming socially and physically active. However, the level of dependency was perceived to be high by the participants, who reported a need for informal support systems to decrease their dependency.

*“The local intermediary is really an angel. She tries to arrange all that’s best for us. It was a very good move from the municipality to hire her.”*

Participants emphasized that they were dependent on others, for example, when something broke down at home. They did not have the money to replace it, or to get a company to repair it. There were few opportunities to get something new or buy it cheap, like in a second-hand shop. Even in a second-hand shop in a city nearby, the products were still seen as quite expensive.

*“My washing machine broke down, and if I had known someone who could help me repair it, then I could have asked him. Maybe it was just some minor thing and easy to fix. Now I had to call a company, and they charge call-out charges and repair costs. I can’t pay all that.”*

Participants also said that being able to speak Dutch and being able to get the right information is necessary to participate in society and help each other. A good information provision, with support about ways of saving money, would be useful. According to the participants, there were many “foreigners” in the municipality, of both Western and non-Western origin, and these people also needed a good source of information. Support for those who do not speak Dutch was also regarded as particularly essential for better integration and participation.

*“I would like to participate in the municipality and help others, but it’s very difficult since I do not speak the language well. The language is the basis. […] I think it would work much better if someone from my country could help me with the Dutch language, instead of going to a very expensive course.”*

To improve their situation, participants would like to have an exchange store in the neighbourhood, where all residents could bring products that they do not need any more and receive points for that, which they can use to obtain other goods. Furthermore, a service exchange system could be developed, where people can offer their services or can ask for a service. Participants saw this as an opportunity for citizens to meet and help each other.

*“Residents in Vaals can give away stuff by putting it in ‘BEST bags’ and put them out in the street. A service then picks up these bags and brings them to a shop for recycled goods in a city nearby. But how can we go there? It is a two-hour trip by bus and we have to pay six euros. If we then want to buy something, which is often quite expensive as well, then it is difficult for us to take it home. Why can’t these items remain in Vaals for our own people?”*

#### Feeling safe

All participants mentioned being dependent on their immediate environment, which makes it important to them that this environment is safe and clean. Regarding social safety, they all reported feeling safe; criminality seemed to be no issue in Vaals. However, most participants reported that they felt unsafe regarding the physical environment. The main road in particular was dangerous for children to cross. The participants said that they had to cross this road several times a day to go to school or to the shops. They reported that since certain adjustments had been made, even more accidents had happened and it took them a lot of time to cross the road. One option to enhance the road safety for pedestrians that was mentioned by participants was that it might be possible to install some warning systems that light up when people want to cross the road or to have crossing guards present during school hours.

*“The pedestrian crossings […] have no zebra markings or traffic lights anymore, and since these adjustments, more accidents have happened. I often have to wait several minutes with the children. I don’t want my children to cross the road by themselves. We have to go on foot, there is no other option. It’s extremely dangerous.”*

A second issue was that they could only use the free recreation facilities in Vaals, such as the playgrounds or the park, because they did not have enough money to take their children to places where they had to pay. Although there were enough facilities, their condition might be improved, especially regarding hygiene. Some participants mentioned they were afraid their children might get ill because of the poor conditions.

*“I don’t have the money to take my children to larger playgrounds or to go out for a day. We have to use the facilities available in Vaals. We’re very happy with these facilities, because there’s a nice park and a lot of decent playgrounds. But they’re not hygienic. There is not only dog poo, but also other trash like cans and plastic bags. That’s not good for my children’s health.”*

#### Being mobile

Participants mentioned that they did not have enough money to buy a car or other motorized means of transport. They needed to use public transport or ask others for help, but they did not always want to rely on others. Public transport was said to be complicated, taking a lot of time and energy. Most of them had to walk for about twenty minutes to get to a bus stop or to the shops in the town centre, and public transport was expensive. They found it unpractical to get their groceries on foot, and especially with small children it took a lot of time.

*“If I have to go to the hospital with my children, it costs me six euros for a ticket and it takes me the whole day. Twenty minutes walking to the bus, half an hour in the bus, transfer, another half hour in the bus and then of course the whole trip back home. If there were a bus stop nearby, that would make it a lot easier.”*

#### Future prospects

In addition to these common themes, participants were also asked about their future prospects. Although they reported finding it difficult to think about their future, they all put the future of their children first. They also indicated they would be willing to participate in society, but wanted support from others to do so.

*“If there is nobody around you who encourages you, by just saying ‘come on’ or ‘let’s go’, then it becomes really difficult. You have to work for a better future, but doing it all on your own is hard.”*

## Discussion

The current study aimed to identify opportunities to improve the current social and health situation of low-SES families and the main difficulties they experience in daily life. Four coherent themes, i.e., meeting each other, helping each other, feeling safe and being mobile, were found to be essential to improve their current situation.

All four themes reflect the desire of the families to be independent and self-resilient (Crane & Heaton, 2008). The money shortage that they experience in daily life makes them dependent on the help of others and their immediate environment. They reported that they did not have the means to go to public places to drink a cup of coffee, to buy things or have them repaired when they break down, to use individual motorized transport or to go to a playground or theme park that charges an entrance fee. Limited mobility appeared to increase the importance of a safe and clean neighbourhood, where people support each other. Another point raised was that most families longed for a sense of belonging (Cooper & Campbell Quick, 2017; Stewart et al., 2009). The participants felt that other citizens were prejudiced against low-SES households, regarding them as inferior, which made them more reticent about engaging in social activities. In addition, the relatively high costs of participating in clubs or societies made them feel more isolated from society. The photographs they took showed that health-related themes did not have a high priority for these families (Van Lenthe, Jansen, & Kamphuis, 2015), as they focused much more on basic resources that could help them cope with their current worries.

The results of this study are in line with Maslow’s Hierarchy of Needs (Maslow, 1943). For the people from lower-SES groups who were included in this photovoice study, the basic needs at the bottom of the pyramid, such as physiological, safety and belongingness needs, appeared largely unsatisfied. These basic needs have to be satisfied first before they can progress to higher-level needs, such as living a healthy life (Huitt, 2007; Van Lenthe et al., 2015). This finding is also in line with the Self-Determination Theory, where the fulfilment of the three primary psychological needs of relatedness, competence and autonomy forms the basis for people to feel satisfied with their lives and enhance their well-being (Deci & Ryan, 2000; Martin & Hill, 2012). The participants in our study felt unable to make their decisions correspond to their basic needs, values and interests, did not feel competent to take control over their lives and lacked a sense of connection to others in their environment.

The results of the present study underline the importance of upstream determinants of health, defined as the more distal factors that have trickle-down effects on more proximal determinants of health. Improving the upstream determinants, such as education, employment opportunities, social/physical context and people’s mindset, is likely to have a positive impact on the feeling of independence, self-resilience and a sense of belonging, as well as on the long-term health outcomes of the population (Australian Medical Association, 2007; Bharmal, Derose, Felician, & Weden, 2015; Di Domenico & Fournier, 2014; Mmari et al., 2014; Steger, Fitch-Martin, Donnelly, & Rickard, 2015; Van Lenthe et al., 2015). In contrast, improving downstream determinants is often compared to “trying to empty the ocean with a thimble”. The importance of focusing on the basic needs as upstream determinants of low-SES families overcoming health inequalities was also stated by Mullainathan and Shafir (2013) and Ball (2015). Most inequalities are related to social inequalities, such as lower education levels, poor housing, no or low-paid jobs and lower income. This results in scarcity which in turn results in a mindset focusing on the question how to survive, causing a cognitive burden. These families have to invest a lot of cognitive energy in their everyday living situation, at the expense of sensible plans and actions related to living healthily (Ball, 2015; Mullainathan & Shafir, 2013). This “scarcity mindset” is a serious risk factor for a lower health status (Fell & Hewstone, 2015). The interaction between the emergence of diseases and challenging environmental circumstances may even result in what has recently been called a “syndemic” (from “synergistic” and “epidemic”) (Singer, Bulled, Ostrach, & Mendenhall, 2017). The accumulation of physical, mental and social problems resulting in such syndemics exacerbates the poverty cycle, and intergenerational transfer is likely to occur, as children grow up with a scarcity mindset (Tiemeijer, 2016; Wagmiller & Adelman, 2009).

Overall, this study revealed the importance of understanding the perceived daily issues low-SES families encounter and their views about their needs. Our approach revealed the importance of first tackling the basic needs of low-SES families before opportunities can arise to enhance their health status. Future studies should focus on meeting these needs, in cooperation with the target group. A long-term approach tackling upstream determinants of the health status of low-SES families may gradually improve their self-resilience and societal sense of belonging, and will eventually contribute to the reduction of socioeconomic health inequalities.

### Strengths and limitations

A strength of this photovoice study was the assistance provided by the local intermediary, who was trusted by the families, which facilitated the recruitment process. Furthermore, the researcher had intensive contact with the families for the relatively long period of four months, which improved their relationship. With regard to future activities, the close contacts that developed among the participating families formed a solid basis to develop activities together with professionals in the municipality of Vaals.

In addition to these strengths, some limitations should be acknowledged. Although a diverse group of families participated, they needed to have a relatively strong commitment, since participation took them considerable effort and time. This may have limited the recruitment of participants in the first place. Although the photovoice methodology may have some attractive features compared to traditional research methods, selection bias might still have occurred, as the families in the worst conditions may not have been willing to participate. While the number of participants was appropriate for a photovoice study (Wang, 1999), the relatively low number of participating families might reduce the generalizability of the results (Catalani & Minkler, 2010).

### Recommendations for research and practice

This study tried to explore the most important needs felt by the participating low-SES families. Our results clearly show that low-SES groups should be involved in research as experts, to find tailored gateways to enhance their situation (Merzel & D’Afflitti, 2003; Movisie, 2012a; Parker, 2003). By participating in the photovoice they became more aware of their needs and converted those into priorities, which enabled them to advocate for it. To increase their participation and commitment, health promotion programmes should focus on their basic needs first, instead of focusing only on promoting health, since healthy living is not their first priority. This can best be achieved by using an integrated approach where low-SES families, local partners and professionals from various disciplines work together to achieve their goals (Lundy, 2010). Structural upstream changes should be made in the existing community that are primarily aimed at the basic needs of the low-SES families; this may eventually reduce socioeconomic health inequalities. This approach can enhance the independence, self-resilience and sense of belonging of low-SES groups, enabling them to improve their own situation (Bosma, 2006). Note that focusing on the upstream determinants implies a longer timeframe for implementation than traditional health promotion interventions aimed at downstream determinants. This approach also forces policy makers, practitioners and researchers to be realistic in their short-term expectations, because immediate health effects cannot be expected. A long-term approach is needed, with sustained political commitment and community involvement.

## Conclusion

This study showed the clear need for involvement and participation among low-SES families. It is essential to first meet their basic needs, such as meeting each other, helping each other, feeling safe and being mobile. The photographs taken by the participants showed that health-related themes were not a high priority for these families. Their focus was on upstream factors related to being able to cope with their current situation. All the basic needs they expressed indicated their desire to be independent and self-resilient and to perceive a sense of belonging. The present study represents a first step towards the development of a community approach by showing the most important themes to focus on.
