# Supplementary material for: Shining light inside the tunnel: using photovoice as a strategy to define the needs for health promotion among families of low socioeconomic status
Source: Int J Qual Stud Health Well-being. 2018 Nov 19;13(1):1542909. doi: 10.1080/17482631.2018.1542909 (PMC6249552; doi:10.1080/17482631.2018.1542909)
Supplement: Supplemental Material [file ZQHW_A_1542909_SM5657.docx]

Appendix 1 Pictures made during the photovoice study

The most illustrative photographs representing the four themes defined in the Photovoice study are depicted below.


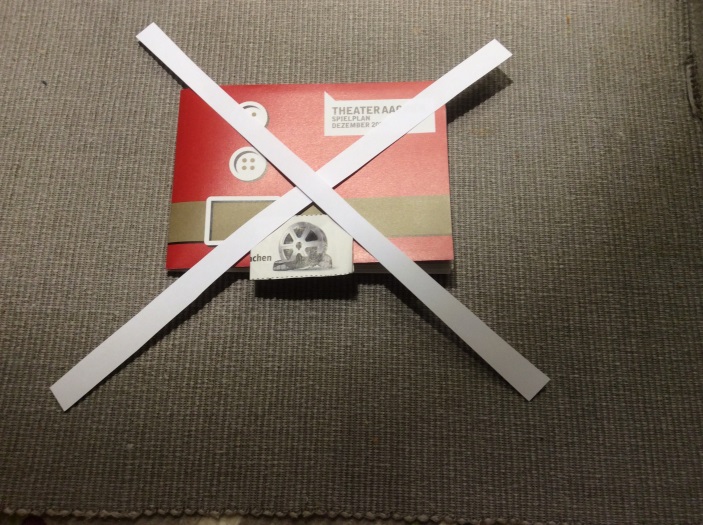
Meeting each other


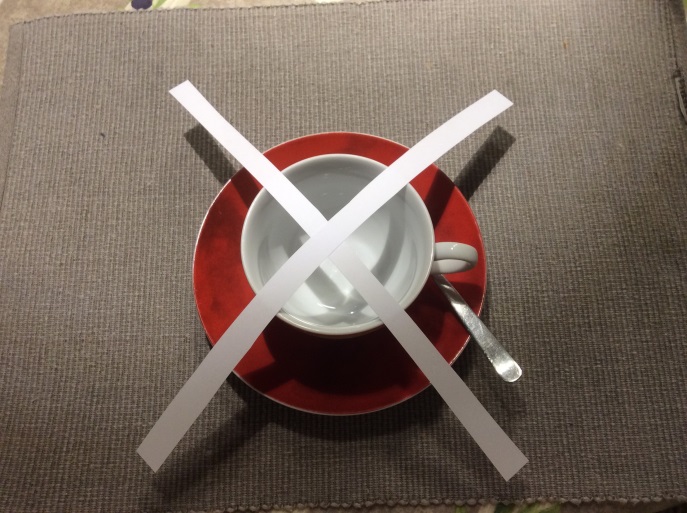


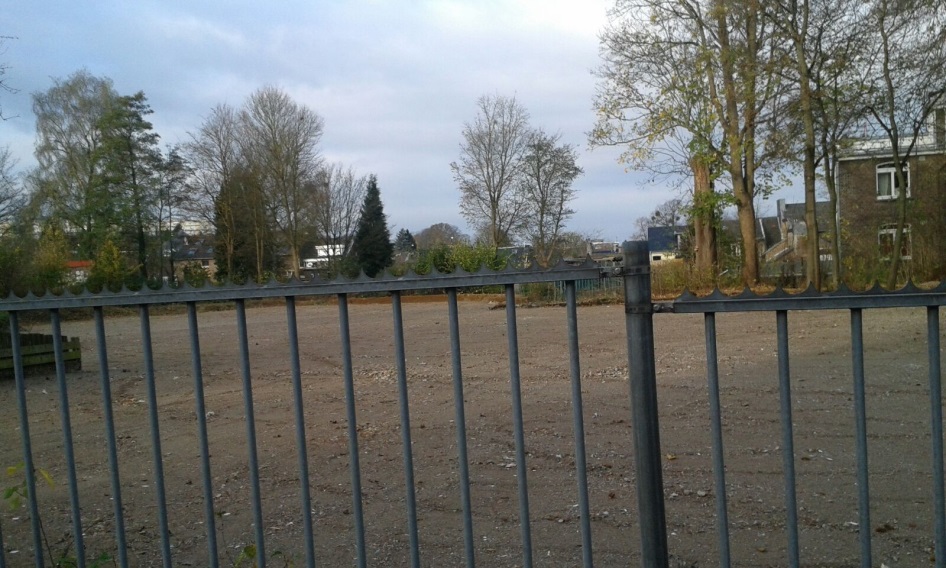


These pictures show the need of the families to have a place in their neighborhood where they can meet each other, organize activities and drink a cup of coffee. The bottom picture shows what could have been a meeting place, but the former elementary school building was demolished.


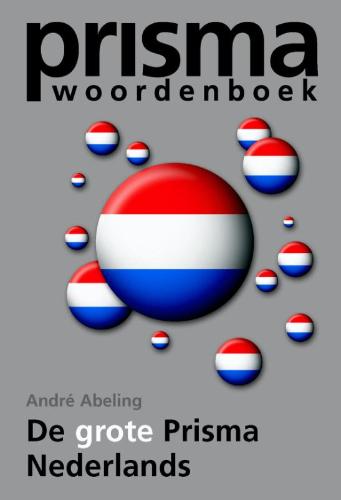
Helping each other


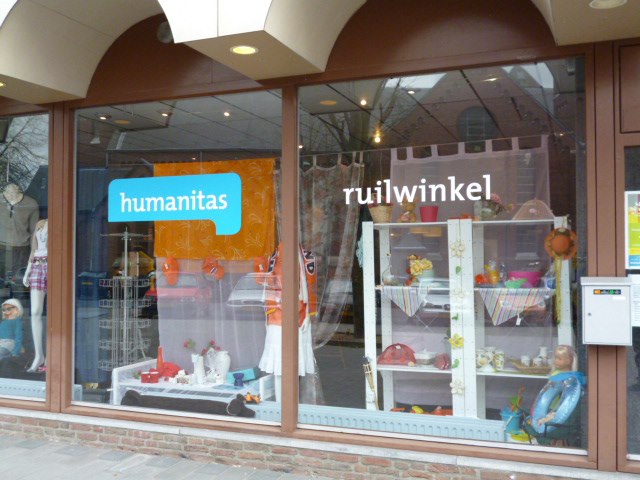

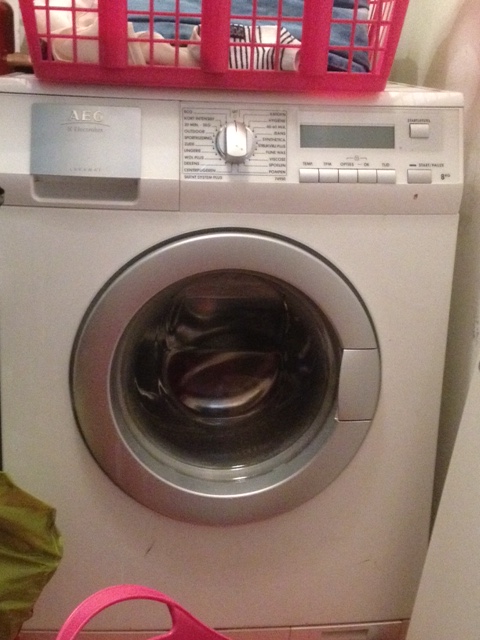

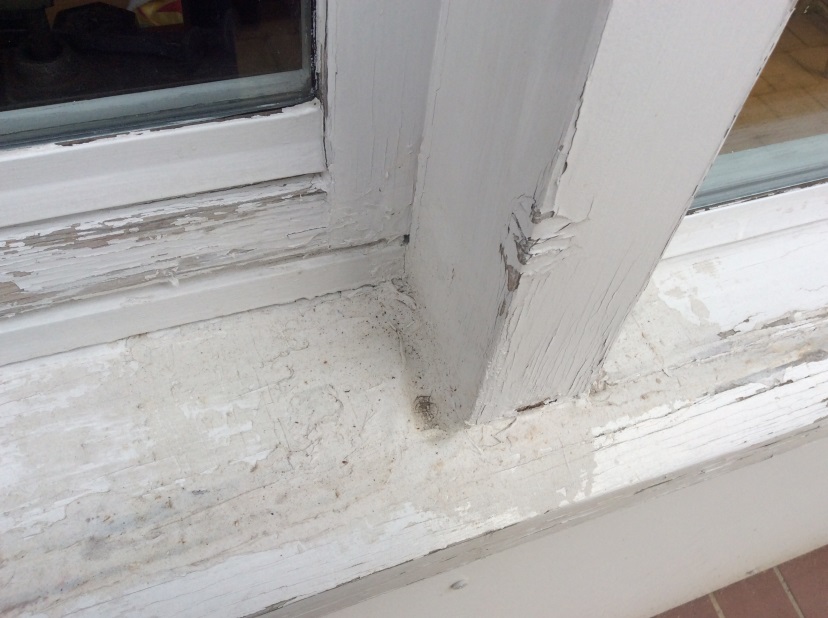


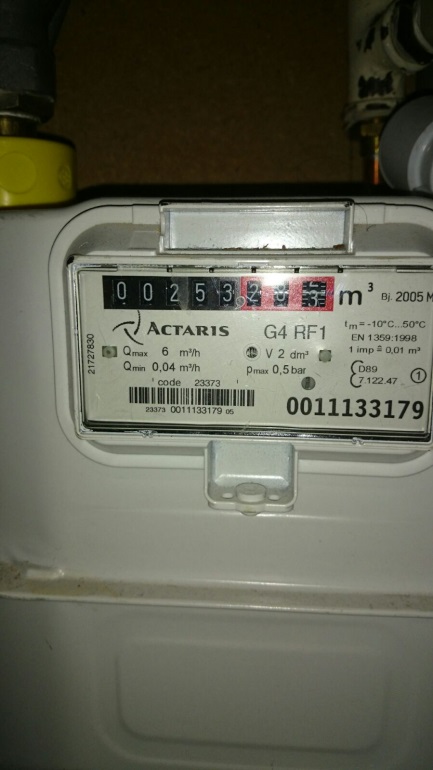

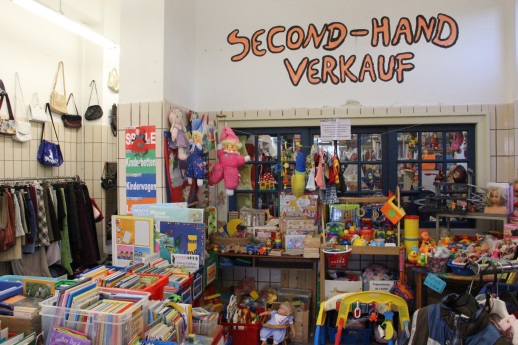

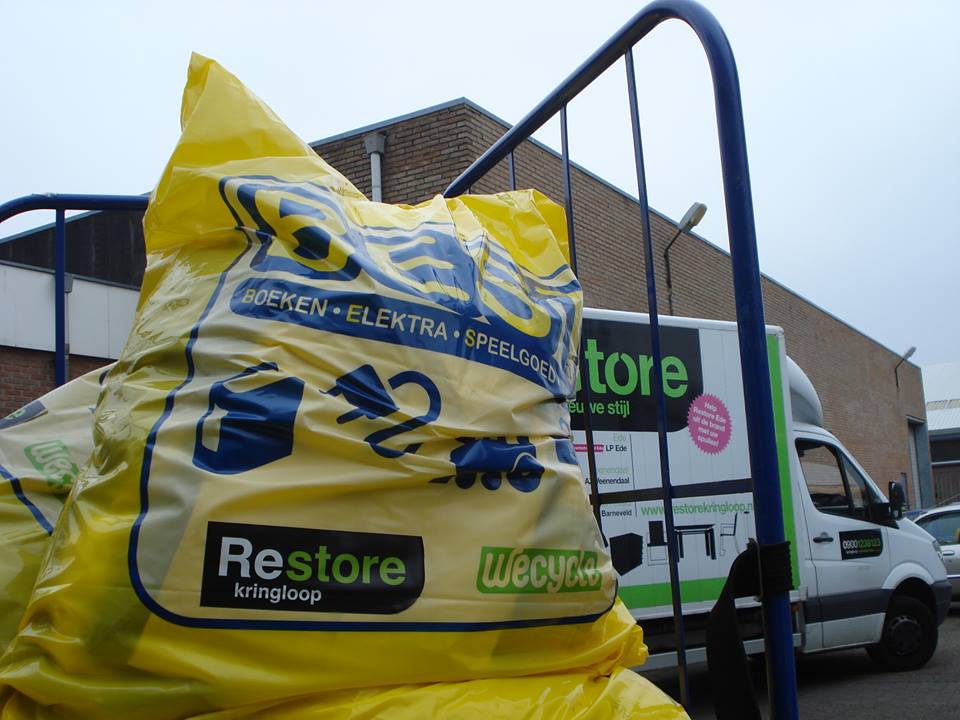


All pictures showed the perceived dependency of the families and the need for more informal support, such as support in learning the Dutch language, help when something breaks down, and cheap or free products that are available for the families such as the products in the yellow bag or in the exchange store.


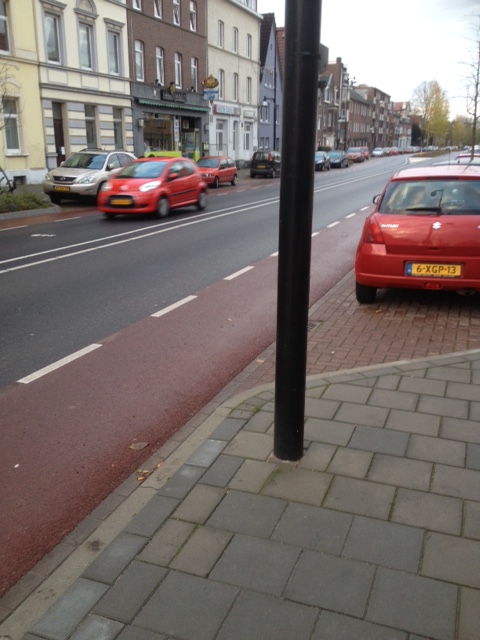
Feeling safe


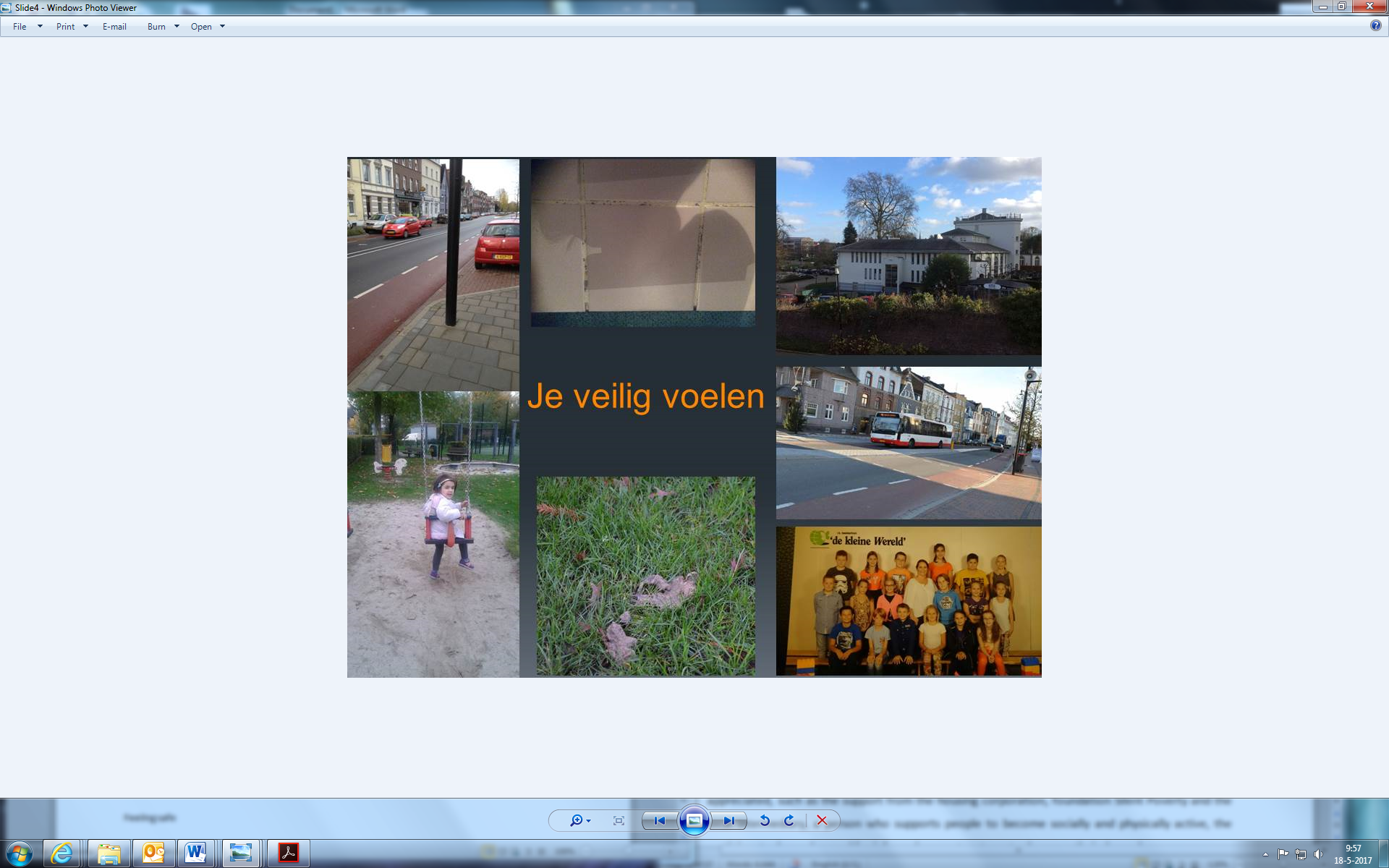

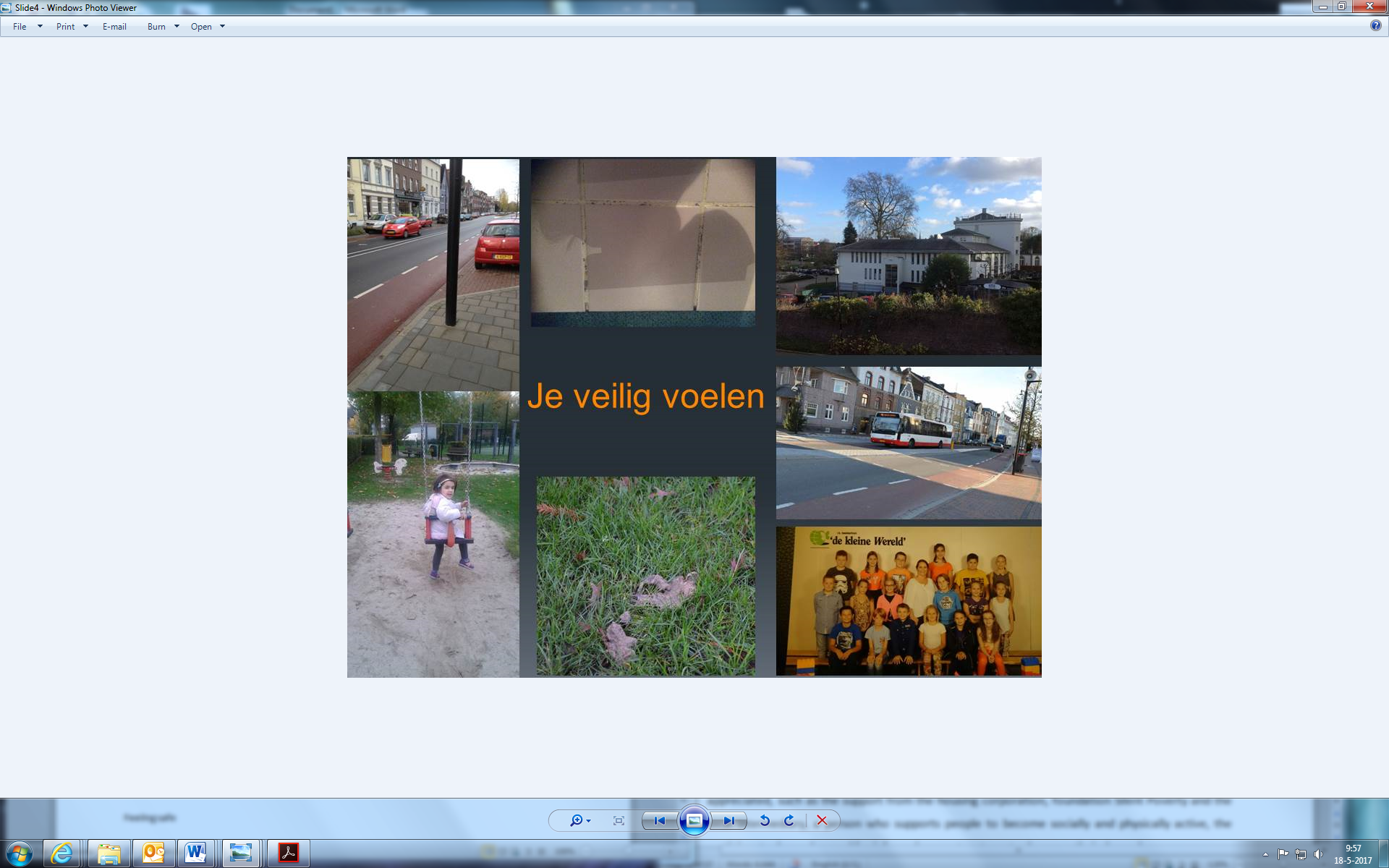


The theme of feeling safe mainly focused on the physical environment. Families mentioned the dangerous road, which they have to cross several times a day to get to the school and the shops. Other photographs show hygiene issues, such as dog poo in the playgrounds.

Being mobile


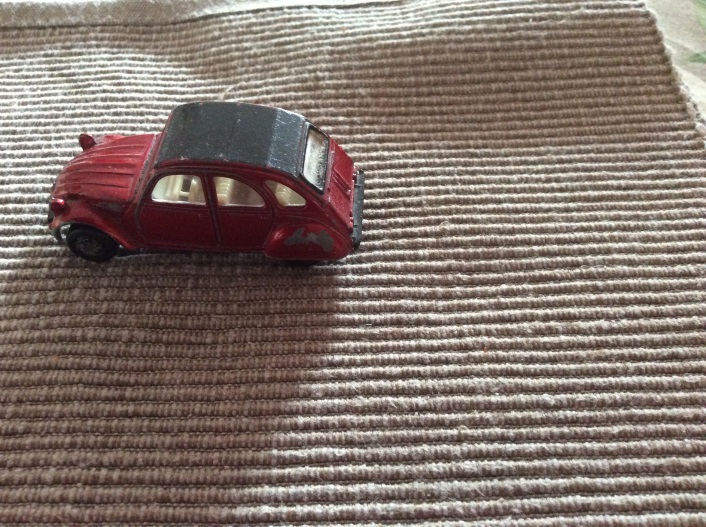

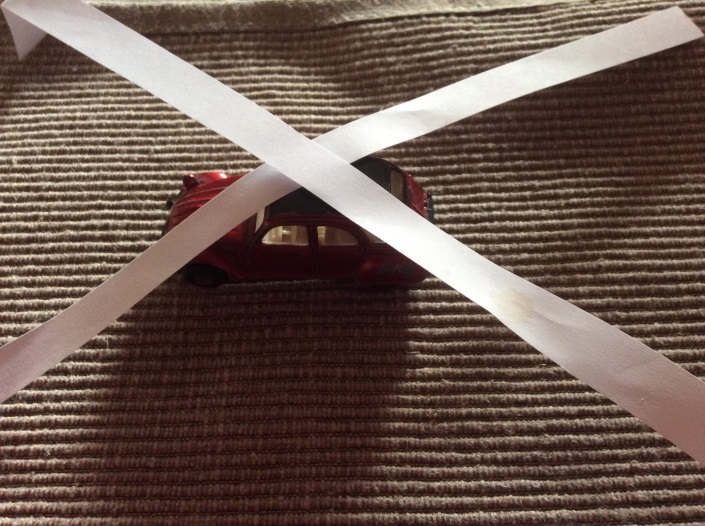


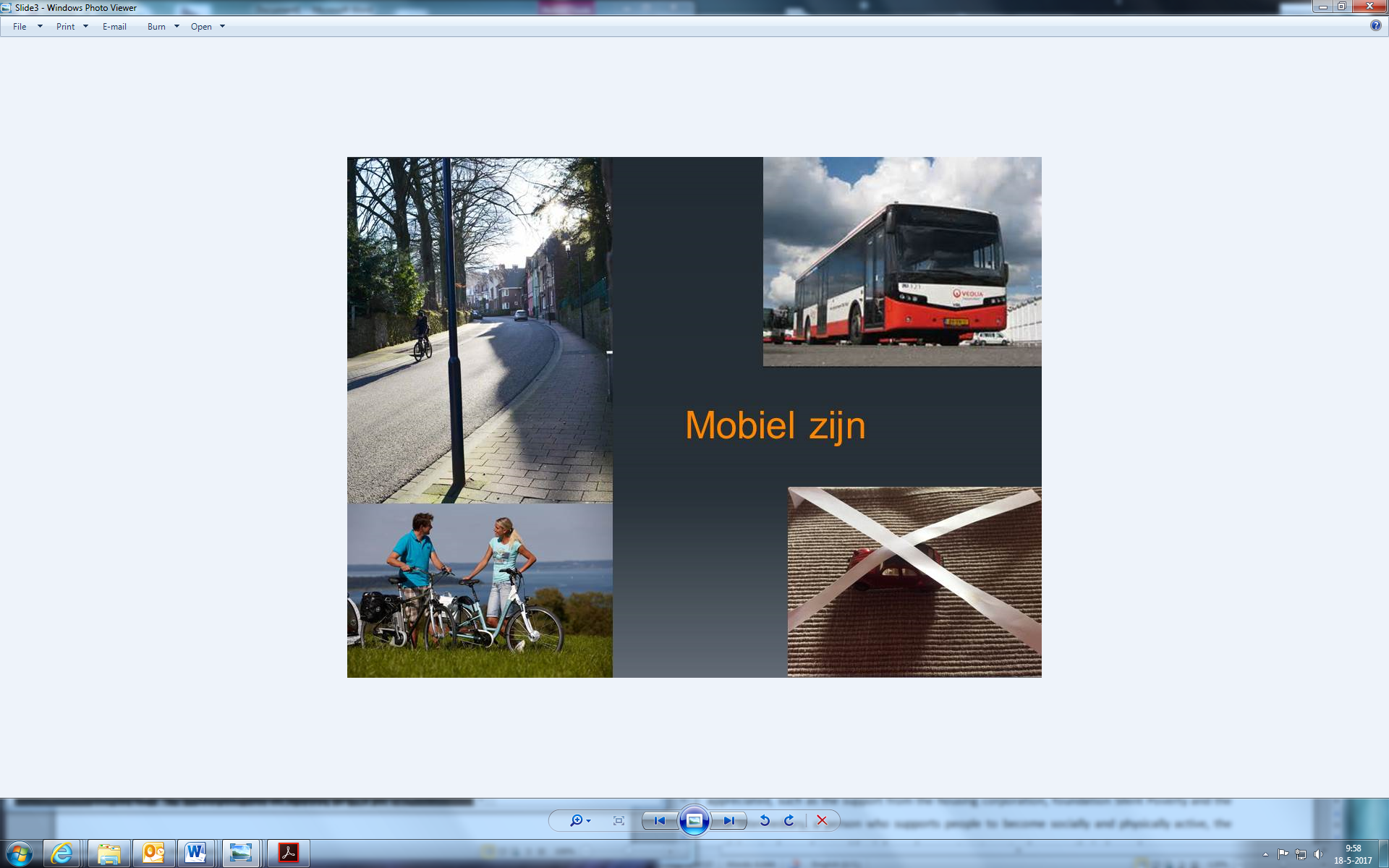


The final theme, being mobile, reflected the limited mobility of low-SES families, because they do not have their own means of transport, and public transport was perceived as difficult. The lower picture shows part of the route that a family member has to walk every day, because there are no bus stops nearby.
